# Novel Use of Valve-in-Valve Transcatheter Mitral Replacement for Severe Prosthetic Stenosis Due to Drug Use-Associated Endocarditis

**DOI:** 10.1016/j.jscai.2022.100498

**Published:** 2022-10-04

**Authors:** Lucas X. Marinacci, Michael C. Gavin, Roger J. Laham

**Affiliations:** a Division of Cardiovascular Medicine, Beth Israel Deaconess Medical Center, Boston, Massachusetts; b Harvard Medical School, Boston, Massachusetts

**Keywords:** mitral stenosis, prosthetic valve endocarditis, valve-in-valve transcatheter mitral valve replacement

## Case presentation

A 37-year-old man with substance use disorder (SUD) and surgical mitral valve replacement (29-mm St. Jude Epic bioprosthesis) for *Staphylococcus aureus* mitral valve endocarditis 5 years prior to presentation complicated by *Streptococcus mitis* prosthetic valve endocarditis requiring a repeat surgical mitral valve replacement (29-mm St. Jude Epic bioprosthesis) 2 years prior to presentation presented with subacute shortness of breath, chest pain, and a syncopal episode in the context of active injection drug use. He had not been on any medications for opiate use disorder for over a year and reported near daily use of cocaine and injection of opiates; however, he had endorsed benefit from buprenorphine-naloxone in the past and a desire to engage in SUD treatment with strong social support.

He had a temperature of 101.6 °F, heart rate of 148 beats/min, blood pressure of 84/55 mm Hg, respiratory rate of 32 breaths/min, and saturation of 92% using pulse oximetry on room air. On examination, he was found to have bilateral crackles and cool extremities, without peripheral edema; he was agitated and unable to follow commands. Because of respiratory distress and the inability to protect his airway, he was intubated and transferred to the cardiac critical care unit, where a pulmonary artery catheter confirmed cardiogenic shock. He was started on inotropes and vasopressors.

**Figure 1:**
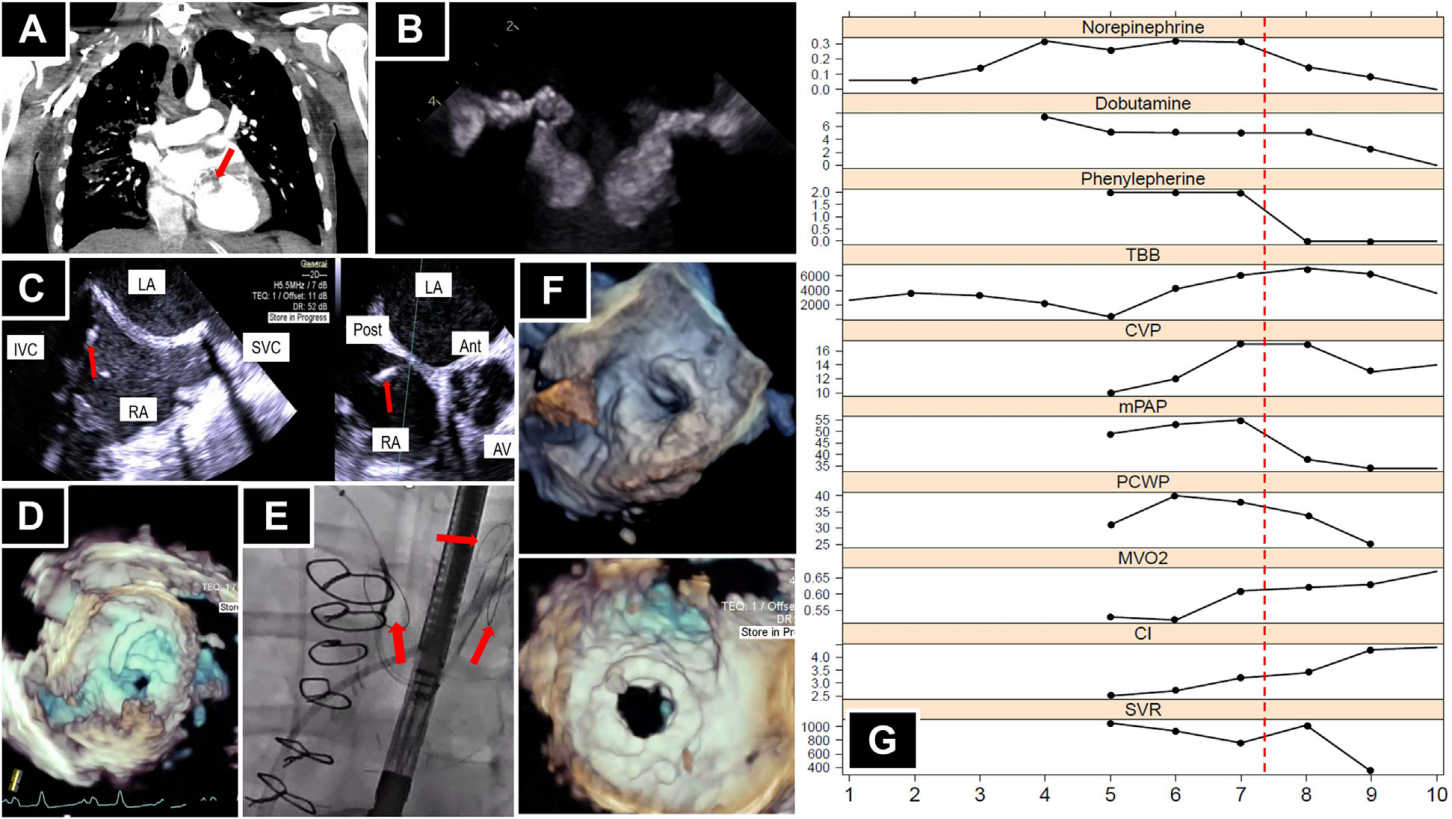
(**A**) Computed tomography scan of the chest with a contrast agent. The arrow indicates bulky hypodensities involving the prosthetic mitral valve apparatus. (**B**) Transesophageal echocardiogram (TEE) demonstrating vegetation on the prosthetic mitral valve leaflets and frame creating significant functional stenosis. (**C**) TEE view of a transeptal puncture needle at the inferior posterior aspect of the fossa ovalis, indicated by the arrow. (**D**) Three-dimensional TEE view of the Agilis sheath flexed into position prior to passing a wire across the mitral valve. (**E**) Fluoroscopic view of deployed Sentinel cerebral protection devices (arrows) via bilateral radial arteries. (**F**) Three-dimensional TEE view of the prosthetic mitral valve before (top) and after (bottom) the procedure. (**G**) Medication doses and hemodynamic data (y-axis) versus days in the intensive care unit (x-axis). The red dotted line indicates the time of valve-in-valve transcatheter mitral replacement. Ant, anterior; AV, aortic valve; CI, cardiac index; CVP, central venous pressure; IVC, inferior vena cava; LA, left atrium; mPAP, mean pulmonary artery pressure; MVO_2_, mixed venous oxygen saturation; PCWP, pulmonary capillary wedge pressure; Post, posterior; RA, right atrium; SVC, superior vena cava; SVR, systemic vascular resistance; TBB, total body fluid balance.

Laboratory examination results were notable for elevation of lactate level, and a toxicology screen was positive for opiates and cocaine. Blood cultures were positive for *S mitis*. Computed tomography angiography of the chest, abdomen, and pelvis demonstrated bulky hypodensities surrounding the mitral valve prosthesis (Figure 1A), septic pulmonary emboli, and splenic infarcts. Transthoracic echocardiography (TTE) demonstrated an ejection fraction of 21%, with inferior and posterior wall motion abnormalities and vegetations on the prosthetic valve apparatus. Transesophageal echocardiography (TEE) demonstrated a well-seated mitral bioprosthesis with echodense vegetations attached to the prosthetic valve leaflets extending into the mitral valve frame, severe stenosis (mean gradient of 16 mm Hg at a heart rate of 77 beats/min), and no abscess (Figure 1B, Supplemental Video 1).

## Intervention

He was deemed a nonsurgical candidate because of recent injection drug use and cardiogenic shock. His blood cultures cleared with initiation of antibiotics. He was initially treated with vancomycin, cefepime, and gentamycin. Because of refractory shock due to severe mitral stenosis, the decision was made to pursue salvage valve-in-valve transcatheter mitral replacement (VIV-TMVR). No dedicated cardiac computed tomography was performed; measurements and sizing decisions were made based on TEE data.

A temporary pacemaker was advanced via the left femoral vein. The right femoral vein was accessed, and a transeptal puncture was made in a posterior-inferior location across the fossa ovalis from the right atrium to the left atrium (Figure 1C, Supplemental Video 2). A medium-curve Agilis NXT sheath (Abbott) was advanced into the left atrium and flexed to line up with the mitral valve, and a pigtail catheter was advanced into the left ventricle (Figure 1D, Supplemental Video 3). Given the risk of embolization, 2 Sentinel cerebral protective devices (Boston Scientific) were deployed, one via the right radial artery to protect the innominate and left carotid arteries and the other via the left radial to protect the left subclavian and, by extension, the left vertebral artery (Figure 1E, Supplemental Video 4).

A 0.035-inch Confida wire (Medtronic) was advanced via the pigtail catheter into the left ventricular apex. A 16F expandable sheath was advanced via the right femoral vein. The septum was dilated using a 14-mm balloon, and the prosthetic valve was dilated using the 24-mm True Balloon (Bard) with rapid right ventricular pacing (Supplemental Video 5). When this failed to relieve stenosis, a 29-mm Sapien 3 valve (Edwards Lifesciences) was positioned using TEE and fluoroscopic landmarks and deployed successfully (Supplemental Video 6).

After deployment, TEE demonstrated significant improvement of mitral stenosis (from a mean gradient of 28 mm Hg at a heart rate of 77 beats/min to a mean gradient of 9 mm Hg at a heart rate of 100 beats/min), with a minimal left ventricular outflow tract (LVOT) gradient despite the presence of a vegetation in the LVOT, a small atrial septal defect with minimal shunting, and no paravalvular leak (Figure1F, Supplemental Video 7). Postprocedural TTE demonstrated a 1.4-cm mass in the LVOT with no gradient, and a mean prosthetic mitral valve gradient of 11 mm Hg at a heart rate of 99 beats/min (Supplemental Video 8). His shock resolved, and after 13 days in the cardiac care unit, the patient was transferred to the floor, where he was started on medications for opiate use disorder (Figure 1G). His antibiotics were narrowed to intravenous ceftriaxone for a 6-week course with 2 weeks of gentamycin for synergy. Repeat blood cultures yielded negative results. He was again evaluated by the cardiac surgery department, which determined that he was at too high risk for a repeat third-time sternotomy. He remained in the hospital for a total of 32 days before he was transferred to another hospital for a second opinion regarding surgical candidacy.

At the second hospital, a repeat TTE and TEE failed to show any vegetation, including in the LVOT, and head and body imaging did not demonstrate any evidence of additional embolic phenomena. Therefore, the decision was made to continue medical management with monitoring and antibiotics without surgery. He completed the course of intravenous antibiotics and was placed on chronic suppressive therapy with oral amoxicillin. He has since undergone surveillance TTE every 3 months, which revealed stable gradients and no vegetations. At 10 months after discharge, the patient has remained compensated from the perspective of heart failure and abstinent from injection drugs, without evidence of relapsing or recurrent infection.

## Discussion

This is the first report, to our knowledge, of the use of VIV-TMVR for the treatment of prosthetic mitral stenosis due to infective endocarditis. Interventional procedures, including transcatheter valve replacement, edge-to-edge repair, and aspiration vegetectomy, are emerging as alternatives to upfront operative management for patients with unstable endocarditis who are deemed poor surgical candidates.^1^, ^2^, ^3^ As this case demonstrates, this may be especially relevant for those with drug use-associated infective endocarditis (DUA-IE).

The incidence of DUA-IE is rising, and the relapse of injection drug use is a primary driver of poor surgical outcomes.^4^ Although the 2020 American Heart Association/American College of Cardiology guidelines do not mention catheter-based interventions for DUA-IE, they recognize that referral to addiction therapy can reduce morbidity and mortality in these patients.^5^ For the subset of patients with DUA-IE who are deemed to be at too high risk for surgery but have lesions that are amenable to catheter-based therapies, successful transcatheter interventions may allow for short-term stabilization and, therefore, additional time to understand, address, and treat underlying SUD. Surgery, if still indicated, can then be reconsidered at a later date after the patient has engaged in addiction treatment and demonstrated the willingness and ability to remain abstinent from unsafe injection practices.

The benefits of immediate hemodynamic stabilization due to transcatheter valve replacement or repair must be balanced with the risk of embolization, obstruction, or seeding of newly implanted prosthetic material. Similarly, the benefits of improved source control or reduction of embolization burden with percutaneous aspiration must be weighed against iatrogenic valve injury. However, if death or severe disability is highly likely without intervention and surgery is not being offered, these options can be reasonably considered within the framework of shared decision making between the multidisciplinary endocarditis team and the patient or their proxy. Therefore, in select cases, an interventional procedure may offer a potential bridge to more definitive treatment and, on rare occasions, be a durable destination therapy in itself.

## Conclusion

Valve-in-valve transcatheter mitral replacement can be considered as salvage therapy for cardiogenic shock caused by severe prosthetic mitral valve stenosis due to endocarditis when surgery is not an immediate option in select candidates. Prospective studies are needed to better define patients who may benefit from transcatheter interventions either as destination therapy or as a bridge to eventual surgery for endocarditis, especially in the setting of injection drug use.

## Supplementary material

Supplemental Video 1 Pre procedure TTE and TEE.

Supplemental Video 2 TEE of transeptal puncture needle at the inferior posterior aspect of the fossa ovalis, indicated by the arrow. IVC: Inferior vena cava; RA: Right atrium; SVC: Superior vena cava; LA: Left atrium; Post: Posterior; Ant: Anterior; AV: Aortic valve.

Supplemental Video 3 Left: 3D TEE of medium curl Agilis sheath advanced to the left atrium via transeptal puncture and flexed to line up with the mitral valve. Right: Fluoroscopy of pigtail catheter advanced into the left ventricle via Agilis sheath.

Supplemental Video 4 Fluoroscopy demonstrating positions of deployed Sentinel cerebral protection devices.

Supplemental Video 5 Fluoroscopy (left) and TEE (right) view of balloon inflation across stenotic prosthetic mitral valve during rapid right ventricular pacing.

Supplemental Video 6 From left to right: Fluoroscopic view of mounted Sapien valve across the dilated prosthetic mitral valve apparatus. TEE view of valve deployment during rapid right ventricular pacing. Post deployment fluoroscopy of VIV-TMVR.

Supplemental Video 7 3D TEE views of the bioprosthetic mitral valve before (left) and after (right) VIV-TMVR.

Supplemental Video 8 TTE parasternal long view demonstrating VIV-TMVR with vegetation in the LVOT.
